# Mechanical variations induce by foot orthoses on calcaneal fracture

**DOI:** 10.1186/1757-1146-7-S1-A9

**Published:** 2014-04-08

**Authors:** T Breard, M Janin

**Affiliations:** 1 Applied Podiatry College, 7 Treguel, 86000 Poitiers, France; 2 Podiatrist, PhD, Clinic, 7 Treguel, 86000 Poitiers, France; 3 Maison médical de Roaillan, 33210 Roaillan, France

## 

This work is meant to quantify the benefits of the foot orthoses [1,2] through the clinical case of a female patient aged 67 who broke her right calcaneum.

**Figure 1:**
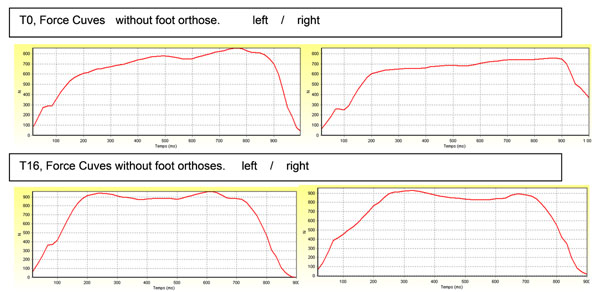
T0, Force Cuves without foot orthose. Left/Right and T16, Force Cuves without foot orthoses. Left/right

To investigate this, we implemented three experiments commonly used during podiatric examinations to assess walking parameters: the passive antepulsion test, the stabilo-baro-podometrie analysis [3,4] and the Latero-Medial Index, measures taken immediately (T0) and after 16 days (T16) wearing plantar orthotics. (figure 1) Foot orthoses, deduced after clinical examination and quantitative analysis of walking, are molded, wisch are supplemented by the addition of specific low stimulations. The results clearly show the benefits on stability. The foot orthoses allows the patient to recover the normal use of the ankle thanks to the positive effects on support and movement of a fractured foot [5]. Therefore, the foot orthoses tends to improve the balance of the fractured foot. Moreover, these positive effects are persistent throughout the time.

In a latter phase, the adjustments carried out on the foot orthoses that modify the foot simulation [3,4], result in the improvement of the assessed parameters (static and dynamic). These variations tend to prove the benefits of the foot orthoses and justify the podiatric approach developed on this clinical case.
